# Risk factors for mortality in ventilator-associated tracheobronchitis: a case-control study

**DOI:** 10.1590/S1679-45082017AO3865

**Published:** 2017

**Authors:** Leonilda Giani Pontes, Fernando Gatti de Menezes, Priscila Gonçalves, Alexandra do Rosário Toniolo, Claudia Vallone Silva, Julia Yaeko Kawagoe, Camila Marques dos Santos, Helena Maria Fernandes Castagna, Marinês Dalla Valle Martino, Luci Corrêa

**Affiliations:** 1 Hospital Israelita Albert Einstein, São Paulo, SP, Brazil.; 2 Universidade Federal de São Paulo, São Paulo, SP, Brazil.

**Keywords:** Bronchitis/mortality, Respiration, artificial, Risk factors, Microbiology, Intensive care units

## Abstract

**Objective:**

To describe the microbiological characteristics and to assess the risk factors for mortality of ventilator-associated tracheobronchitis in a case-control study of intensive care patients.

**Methods:**

This case-control study was conducted over a 6-year period in a 40-bed medical-surgical intensive care unit in a tertiary care, private hospital in São Paulo, Brazil. Case patients were identified using the Nosocomial Infection Control Committee database. For the analysis of risk factors, matched control subjects were selected from the same institution at a 1:8.8 ratio, between January 2006 and December 2011.

**Results:**

A total of 40 episodes of ventilator-associated tracheobronchitis were evaluated in 40 patients in the intensive care unit, and 354 intensive care patients who did not experience tracheobronchitis were included as the Control Group. During the 6-year study period, a total of 42 organisms were identified (polymicrobial infections were 5%) and 88.2% of all the microorganisms identified were *Gram*-negative. Using a logistic regression model, we found the following independent risk factors for mortality in ventilator-associated tracheobronchitis patients: Acute Physiology and Chronic Health Evaluation I score (odds ratio 1.18 per unit of score; 95%CI: 1.05-1.38; p=0.01), and duration of mechanical ventilation (odds ratio 1.09 per day of mechanical ventilation; 95%CI: 1.03-1.17; p=0.004).

**Conclusion:**

Our study provided insight into the risk factors for mortality and microbiological characteristics of ventilator-associated tracheobronchitis.

## RESUMO

**Objetivo:**

Descrever as características microbiológicas e avaliar os fatores de risco para mortalidade na traqueobronquite associada à ventilação mecânica em um estudo caso-controle de pacientes de terapia intensiva.

**Métodos:**

Estudo realizado ao longo de 6 anos em uma unidade de terapia intensiva médico-cirúrgica de 40 leitos, em um hospital privado e de nível terciário em São Paulo, Brasil. O Grupo Caso foi identificado usando o banco de dados da Comissão de Controle de Infecção Hospitalar. O Grupo Controle foi pareado na proporção de 1:8,8 entre janeiro de 2006 e dezembro de 2011.

**Resultados:**

Quarenta episódios de traqueobronquites associadas à ventilação foram avaliados em 40 pacientes na unidade de terapia intensiva, e 354 pacientes não apresentaram traqueobronquite Grupo Controle. Foram identificados 42 microrganismos (dos quais 5% foram infecções polimicrobianas), sendo que 88,2% de todos os microrganismos eram bactérias *Gram*-negativas. Usando um modelo de regressão logística, encontramos os seguintes fatores de risco independentes para mortalidade em pacientes com traqueobronquites associadas à ventilação: pontuação da *Acute Physiology and Chronic Health Evaluation* I (*odds ratio* 1,18 por uma unidade de pontuação; IC95%: 1,05-1,38; p=0,01) e duração da ventilação mecânica (*odds ratio* 1,09 por dia de ventilação mecânica; IC95%: 1,03-1,17; p=0,004).

**Conclusão:**

Nosso estudo forneceu informações sobre os fatores de risco para mortalidade e características microbiológicas da traqueobronquite associada à ventilação mecânica.

## INTRODUCTION

Lower respiratory tract infections are an important cause of morbidity and mortality in critical care patients. Although ventilator-associated pneumonia (VAP) has been at the center of scientific investigation for several years, much less attention was given to ventilator-associated tracheobronchitis (VAT), especially concerning mortality risk factors. This fact may be attributed to the reluctance of many authorities to consider VAT as an independent clinical entity.^1,2^

## OBJECTIVE

To describe the microbiological characteristics and the risk factors for mortality of patients with ventilator-associated tracheobronchitis in an intensive care unit.

## METHODS

This case-control study was conducted over a 6-year period in a 40-bed medical-surgical intensive care unit (ICU) with the same physical layout as that in a tertiary care, private hospital in São Paulo, Brazil. The study was reviewed and approved by the hospital Research Ethics Committee, under protocol number CAAE: 04355112.1.0000.0071.

The patients were identified using the Infection Control Committee database. For the analysis of risk factors, matched control subjects were selected from the same organization at a 1:8.8 ratio, between January 2006 and December 2011. The control subjects were matched for six characteristics: sex, age (10-year maximum difference), time of mechanical ventilation (20-day maximum difference), use of tracheostomy, use of bi-level positive pressure airway (BiPAP), and number of days in the intensive care unit (20-day maximum difference). The medical records of all case patients and control subjects were reviewed.

Ventilator-associated tracheobronchitis was classified according to the Centers for Disease Control and Prevention (CDC) definitions.^3^ Microorganisms were initially identified by Microbiology Department of the organization, using quantitative cultures of tracheal aspirates Colony Forming Units (CFU) (≥1x10^6^CFU/mL) or bronchoscopic specimens (≥1x10^4^CFU/mL).

All statistical analyses were performed using the R Project for Statistical Computing, version 3.1.1. Categorical variables were analyzed using the χ^2^ test. Continuous variables were compared using the Wilcoxon-Mann-Whitney test. Univariate analysis was conducted for potential risk factors for mortality in tracheobronchitis associated with mechanical ventilation, and variables with p value of less than 0.15 were included in the multivariate model. A 2-tailed p value of 0.05 or less was considered statistically significant.

## RESULTS

Between January 2006 and December 2011, 40 episodes of VAT were evaluated in 40 patients in the intensive care unit, and 354 intensive care patients who did not experience tracheobronchitis were included as the Control Group. Demographic and clinical characteristics of the Case and Control Groups are presented in table 1.

**Table 1:** Univariate analysis of clinical and demographic characteristics of 40 episodes of ventilator-associated tracheobronchitis and 354 matching control subjects

Variable	Case Group (n=40)	Control Group (n=354)	p value
Mean age (range), years	67.8 (24-94)	62.8 (16-100)	0.133
Male sex, (%)	62.5	62.7	0.979
Cardiovascular disease, (%)	60	53.4	0.428
Pulmonary disease, (%)	17.5	9.3	0.111
Cancer disease, (%)	17.5	20.6	0.642
Surgical patient, (%)	40	27.4	0.098
Mean APACHE I score, range	21.9 (0-44)	22 (7-48)	0.904
Duration of mechanical ventilation, mean (range), days	21.9 (4-76)	11 (1-76)	<0.001
Duration of BiPAP, mean (range), days	2.6 (0-21)	1.6 (0-31)	0.132
Duration of tracheostomy, mean (range), days	17.3 (0-92)	4.8 (0-117)	<0.001
Length of stay at intensive care unit, mean (range), days	32 (5-88)	16.4 (1-251)	<0.001
Length of hospital stay, mean (range), days	66.3 (15-265)	34.9 (1-623)	<0.006

Categorical variables were analyzed using the χ^2^ test and continuous variables were compared using the Wilcoxon-Mann-Whitney test.

APACHE I: Acute Physiology and Chronic Health Evaluation I; BiPAP: bilevel positive pressure airway.

During the 6-year study period, 42 microorganisms were identified (polymicrobial infections were 5%), and 88.2% of all the microorganisms identified were *Gram*-negative. The etiologic agents of the VAT episodes are shown in table 2.

**Table 2:** Etiology of 40 episodes of ventilator-associated tracheobronchitis

Agents	Infections (%)
*Pseudomonas aeruginosa*	31.0
*Klebsiella pneumoniae*	19.0
*Serratia marcescens*	7.0
*Staphylococcus aureus*	7.0
*Enterococcus faecalis*	4.80
*Enterobacter aerogenes*	4.80
*Acinetobacter baumannii*	4.80
*Stenotrophomonas maltophilia*	4.80
*Elizabethkingia meningoseptica*	4.80
*Achromobacter xylosoxidans*	2.4
*Citrobacter koseri*	2.4
*Providencia stuartii*	2.4
*Burkholderia pickettii*	2.4
*Acinetobacter lwoffii*	2.4

Total	100

We found very high levels of antibiotic resistance: 78.8% of the *Staphylococcus aureus* isolates were methicillin-resistant; and 52.5% of *Pseudomonas aeruginosa* isolates, and 48.7% of *Klebsiella pneumoniae* isolates were carbapenem-resistant. The rate of mortality due to VAT was 42.5%. Using a conditional logistic regression model (Table 3), we found the following independent risk factors for mortality in VAT patients: Acute Physiology and Chronic Health Evaluation I (APACHE I) score (odds ratio – OR: 1.18 per unit of score; 95% confidence interval – 95%CI: 1.05-1.38; p=0.01), and duration of mechanical ventilation (OR: 1.09 per day of mechanical ventilation; 95%CI: 1.03-1.17; p=0.004).

**Table 3:** Univariate and multivariate analysis of risk factors for mortality from ventilator-associated tracheobronchitis

Variable	Univariate analysis		Multivariate analysis	
	
	OR	95%CI	OR	95%CI
Mean age (range), years	1.05	1.01-1.10	-	-
Male sex	0.76	0.20-2.81	-	-
Cardiovascular disease	1.41	0.39-5.33	-	-
Pulmonary disease	4.37	0.80-33.99	-	-
Intestinal disease	1.43	0.23-8.74	-	-
Cancer disease	0.17	0.01-1.19	-	-
Surgical patient	1.66	0.46-6.15	-	-
APACHE I mean (range)	1.14	1.03-1.29	1.18	1.05-1.38
Duration of mechanical ventilation, mean (range), days	1.09	1.02-1.17	1.09	1.03-1.17
Duration of BiPAP, mean (range), days	1.08	0.96-1.25	-	-
Duration of tracheostomy, mean (range), days	1.05	1.01-1.10	-	-
Length of stay at intensive care unit, mean (range), days	1.04	1.01-1.08	-	-
Length of hospital stay, mean (range), days	0.99	0.97-1.00	-	-

Statistical test: conditional logistic regression model.

OR: odds ratio; 95%CI: 95% confidence interval; APACHE I: Acute Physiology and Chronic Health Evaluation; BiPAP: bilevel positive pressure airway.

## DISCUSSION

Ventilator-associated tracheobronchitis is recognized as a frequent complication of mechanical ventilation with rates ranging from 3.7 to 11.5%, according to the literature.^4,5^ In addition, more recent data suggest that VAT may contribute to the need for longer stays at the intensive care unit and the need for a longer duration of mechanical ventilation, as demonstrated in our study. However, there is controversy in the literature regarding whether increased mortality is associated with VAT.^6^

The crude mortality in the Case Group was 42.5% similar from that found in the literature, which ranged from 21 to 55%.^6,7^ The APACHE I score and duration of mechanical ventilation were the independent risk factors for mortality in our study, as already demonstrated in other studies on VAP.^8^ In regard to APACHE I scores, for every increase by one unit in the score, there was an increase by 18% in the risk of death. As to duration of mechanical ventilation, for every increase by 1 day in mechanical ventilation, there was an increase by 9% in the risk of death. Information is lacking regarding the risk factors for mortality in VAT, perhaps because the subjective components within the VAT definition and diagnosis may impact the reliability and accuracy of case identification. In addition, the same risk factors for mortality in VAT and VAP could reinforce the hypothesis that VAT is an intermediate stage between colonization of the upper airways and VAP.^9^

Regarding the etiologic agents of VAT, *Gram*-negative pathogens were the most common cause in many studies, accounting for more than 60% of isolates, similar to that found in our study (88.2%). Over the past 5 years, the incidence of VAT caused by multidrug resistant pathogens, such as carbapenem-resistant *Gram*-negative bacilli (*e.g., P. aeruginosa*, *Acinetobacter baumannii*, and *K. pneumoniae*) and methicillin-resistant *S. aureus*, has increased.^10^

This study has notable limitations. First, we cannot exclude with certainty the possibility that some of our patients in the Case Group were misclassified, because we did not routinely perform lung computed tomography scans searching for occult infiltrates. Second, this is a retrospective observational study, and other unmeasured factors might have occurred coincidentally to the period. Third, data from a single unit and the small sample size limit the generalizability of findings.

## CONCLUSION

Our study provided insight into the risk factors for mortality and microbiological characteristics of ventilator-associated tracheobronchitis over a 6-year study. Further studies on ventilator-associated tracheobronchitis, including risk factors for mortality, are necessary to define the best practices.
